# NAT10 as a central node in cancer biology: integrating epitranscriptomic regulation, metabolic reprogramming, and immune modulation

**DOI:** 10.3389/fimmu.2026.1845155

**Published:** 2026-06-05

**Authors:** Wentao Bo, Ying Yi, Biao Zhao, Hang Dong

**Affiliations:** 1 Department of Hepatopancreatobiliary Surgery, Sichuan Clinical Research Center for Cancer, Sichuan Cancer Hospital and Institute, Sichuan Cancer Center, University of Electronic Science and Technology of China, Chengdu, China; 2 Musculoskeletal Cancer Surgery Department, Sichuan Clinical Research Center for Cancer, Sichuan Cancer Hospital & Institute, Sichuan Cancer Center, University of Electronic Science and Technology of China, Chengdu, China; 3 Department of Integrated Traditional Chinese and Western Medicine, Sichuan Clinical Research Center for Cancer, Sichuan Cancer Hospital and Institute, Affiliated Cancer Hospital of University of Electronic Science and Technology of China, Chengdu, China; 4 Department of General Internal Medicine, Sichuan Clinical Research Center for Cancer, Sichuan Cancer Hospital and Institute, Affiliated Cancer Hospital of University of Electronic Science and Technology of China, Chengdu, China

**Keywords:** ac4C, epitranscriptomics, metabolic reprogramming, NAT10, therapy resistance

## Abstract

N-acetyltransferase 10 (NAT10), the sole known mRNA N4-acetylcytidine (ac4C) writer, has emerged as a central regulator of cancer adaptation. Beyond its canonical role in RNA acetylation, NAT10 integrates environmental stress signals—including hypoxia, metabolic imbalance, and inflammatory cues—with epitranscriptomic remodeling to sustain malignant progression. Through selective ac4C deposition on mRNA and tRNA, NAT10 enhances transcript stability, amplifies translational output, and reinforces oncogenic signaling networks. These molecular effects converge on key adaptive programs, including glycolytic reprogramming, pentose phosphate pathway activation, amino acid biosynthesis, DNA damage repair, drug efflux, vascular remodeling, and immune suppression. Importantly, NAT10 operates within feedback circuits linking HIF-1α, Wnt/β-catenin, YAP1, and VEGFA(Vascular Endothelial Growth Factor A) signaling, thereby transforming transient stress responses into sustained adaptive states. This epitranscriptomic reinforcement underlies tumor plasticity and contributes to resistance against chemotherapy, targeted therapy, and immune checkpoint blockade. Emerging pharmacological strategies targeting NAT10, particularly in rational combination regimens, highlight its translational potential. However, key questions remain regarding ac4C reader proteins, substrate specificity, and context-dependent functions. In this review, we propose an integrated model in which NAT10 functions as a central adaptive node coupling RNA regulation, metabolic plasticity, and microenvironmental remodeling, and discuss future directions for exploiting this axis in precision oncology.

## Highlights

- NAT10 functions as a central adaptive node integrating RNA acetylation, metabolism, and immune remodeling.
- ac4C-mediated transcript stabilization amplifies oncogenic signaling and tumor plasticity.
- Targeting NAT10 may overcome chemotherapy, targeted therapy, and immunotherapy resistance.

## 1 Introduction

Over the past decade, the field of epitranscriptomics has transformed our understanding of gene regulation by revealing that RNA molecules are extensively and dynamically modified. While N6-methyladenosine (m6A) initially dominated the field, growing evidence has highlighted additional modifications that fine-tune RNA fate. Among them, N4-acetylcytidine (ac4C) has emerged as a structurally conserved and biologically meaningful RNA modification. Originally identified in tRNA and rRNA, ac4C was historically considered a static structural modification contributing to translational fidelity. However, the discovery that ac4C is also present in mRNA—and dynamically regulated—marked a conceptual shift. Transcriptome-wide mapping studies demonstrated that ac4C enhances mRNA stability and translation efficiency, thereby directly influencing protein output (1–4). Unlike many RNA modifications whose functions remain partially ambiguous, ac4C shows relatively consistent effects on transcript stability and translational enhancement, positioning it as a potent regulator of gene expression amplitude. The recognition of ac4C as a dynamic and regulatory mRNA modification has prompted increasing investigation into its enzymatic machinery and disease relevance, particularly in cancer (5).

NAT10 is a member of the N-acetyltransferase (NAT) family, a group of enzymes responsible for acetylating proteins, RNA, and other substrates (6, 7). While NAT10 has been extensively studied for its role in RNA ac4C modification in cancer, other family members, including NAT8L, have also been linked to tumor progression and metabolic adaptation, suggesting that multiple NAT enzymes may contribute to cancer cell plasticity and adaptation (8–10). To date, N-acetyltransferase 10 (NAT10) remains the only known enzyme responsible for catalyzing ac4C deposition on mRNA. Originally characterized as a nucleolar acetyltransferase involved in rRNA processing and tRNA modification, NAT10 has since been redefined as a multifunctional RNA-modifying enzyme with broad transcriptomic influence. Structurally, NAT10 contains an acetyltransferase domain and RNA-binding regions that enable selective recognition and modification of target transcripts. In mRNA, ac4C deposition by NAT10 enhances transcript stability, promotes translation, and safeguards specific oncogenic transcripts from degradation (11–13). Beyond mRNA, NAT10-mediated tRNA acetylation further supports translational efficiency, particularly under conditions of cellular stress. Importantly, NAT10 activity is not constitutive. Its expression and function are modulated by oncogenic signaling pathways, hypoxia, inflammatory stimuli, and post-translational modifications. Recent evidence further demonstrates that NAT10 can participate in phase-separated nuclear condensates, suggesting spatial regulation of RNA processing. These findings collectively indicate that NAT10 operates within highly regulated signaling networks rather than functioning as a simple housekeeping enzyme.

Accumulating evidence across diverse tumor types—including gastric, bladder, colorectal, prostate, pancreatic, breast, lung, and hematologic malignancies—demonstrates that NAT10 overexpression is consistently associated with aggressive phenotypes characterized by enhanced metastasis, metabolic rewiring, immune suppression, and therapeutic resistance. Mechanistically, NAT10-mediated N4-acetylcytidine (ac4C) modification stabilizes transcripts encoding key regulators of glycolysis and metabolic flux, including lactate dehydrogenase A (LDHA), phosphofructokinase, muscle type (PFKM), and glucose-6-phosphate dehydrogenase (G6PD), as well as transcripts involved in DNA damage repair, drug efflux, epithelial–mesenchymal transition (EMT), cytoskeletal dynamics, adhesion, extracellular matrix remodeling, immune modulation, and checkpoint-associated pathways (14–18). Through these coordinated targets, NAT10 simultaneously influences multiple hallmarks of cancer. Rather than functioning within a single linear signaling cascade, NAT10 integrates RNA stability and translational control with metabolic adaptation and tumor microenvironment remodeling, forming a coordinated adaptive program that promotes tumor plasticity. Moreover, emerging studies reveal extensive crosstalk between ac4C and other RNA modifications, alongside functional interactions between NAT10 and chromatin-associated regulators. These multilayered regulatory connections further support the concept that NAT10 acts as a systems-level orchestrator of malignant progression and adaptive resilience.

In light of these advances, we propose a conceptual framework in which NAT10 functions not merely as an RNA acetyltransferase, but as a central integrative node in cancer biology. Rather than acting as a modification-specific enzyme, NAT10 serves as a systems-level regulator that links RNA stability and translational control with metabolic plasticity and immune remodeling (19, 20). By coordinating epitranscriptomic reprogramming with metabolic rewiring and microenvironmental adaptation, NAT10 enables tumor cells to dynamically transition between proliferative, invasive, and therapy-resistant states. In this review, we synthesize recent mechanistic and translational studies to define the molecular architecture of NAT10-mediated ac4C regulation, elucidate how NAT10 reshapes metabolic and immune programs, integrate these findings into a unified adaptive network model, and evaluate emerging therapeutic strategies targeting NAT10 and its downstream vulnerabilities. Through this integrative perspective, we aim to reposition NAT10 from a modification-centric enzyme to a master regulator of cancer adaptability, highlighting both fundamental mechanistic insights and clinically actionable opportunities.

## 2 Molecular foundations of NAT10 signaling

To understand how NAT10 operates as a systems-level regulator in cancer, it is essential to first delineate its molecular architecture and upstream regulatory mechanisms. NAT10 activity is shaped not only by its intrinsic catalytic capacity but also by multilayered regulation at transcriptional, post-translational, and spatial levels. Together, these mechanisms position NAT10 as a dynamically controlled node responsive to oncogenic and environmental cues.

### 2.1 NAT10 structure and catalytic functions

NAT10 is a member of the GCN5-related N-acetyltransferase (GNAT) superfamily and contains a conserved acetyltransferase domain that catalyzes the transfer of an acetyl group from acetyl-CoA to cytidine residues, generating N4-acetylcytidine (ac4C) (21). This chemical modification alters base pairing properties and enhances RNA structural stability. Unlike several epitranscriptomic writers that rely on multi-protein complexes for substrate targeting, NAT10 appears capable of directly modifying RNA through its catalytic core, although accumulating evidence suggests that substrate selectivity is influenced by RNA structure and protein interactions rather than simple sequence motifs alone. Originally characterized for its roles in rRNA and tRNA modification, NAT10 has since emerged as the principal mRNA ac4C writer. In tRNA, ac4C enhances codon–anticodon pairing stability and translational fidelity, supporting efficient protein synthesis (22). In mRNA, ac4C deposition increases transcript half-life and promotes ribosome engagement, thereby amplifying protein output. This dual substrate spectrum enables NAT10 to coordinate regulation at two distinct yet interconnected layers—transcript abundance and translational capacity—providing a mechanistic basis for its broad regulatory impact.

Although a canonical RNA-binding domain has not been fully structurally resolved, biochemical and transcriptome-wide mapping studies indicate that NAT10 preferentially associates with structured RNA regions, particularly within coding sequences. ac4C sites are enriched in coding regions and correlate with enhanced mRNA stability and translational efficiency. In contrast to m6A, which typically exerts its effects through dedicated reader proteins, ac4C appears to influence RNA fate through more direct physicochemical mechanisms. Nonetheless, emerging evidence suggests functional crosstalk between ac4C and m6A pathways, pointing to layered epitranscriptomic coordination rather than isolated modification events. The biological consequences of ac4C deposition differ depending on RNA class. On mRNA, ac4C selectively reinforces transcripts encoding metabolic enzymes, DNA repair factors, adhesion molecules, and immune regulators—thereby sustaining oncogenic programs at the transcript-specific level. On tRNA, ac4C enhances global translational resilience, potentially enabling rapid adaptive protein synthesis under stress. Intriguingly, tRNA acetylation may also bias translation toward transcripts enriched in particular codon usages, coupling codon composition to adaptive proteomic output (23–25). Through this dual system of transcript-specific stabilization and translational-system tuning, NAT10 functions not as a simple on–off regulator but as a quantitative modulator of gene expression amplitude—a property particularly advantageous for tumor cells navigating fluctuating microenvironmental pressures.

Although NAT10 is generally recognized as the principal writer of ac4C in mRNA, the determinants that guide its target selectivity remain incompletely understood. Transcriptome-wide ac4C profiling has shown that ac4C is enriched within the coding transcriptome and can promote mRNA stability and translation efficiency, supporting the view that NAT10-dependent ac4C deposition is not completely random (26, 27). Current evidence suggests that NAT10 target selection may be influenced by local RNA structure, sequence context, transcript abundance, RNA accessibility, and subcellular localization. Unlike the m6A system, in which writer complexes and reader proteins provide relatively well-defined layers of specificity, NAT10-dependent ac4C deposition may rely more heavily on RNA structural features and interactions with RNA-binding proteins or co-regulatory factors (26, 28). In cancer cells, many reported NAT10 targets encode proteins involved in metabolism, DNA damage repair, drug efflux, adhesion, and oncogenic signaling, including collagen type V alpha 1 chain (COL5A1), kinesin family member 23 (KIF23), multidrug resistance protein 1 (MDR1, also known as ABCB1), breast cancer resistance protein (BCRP, also known as ABCG2), and other cancer-associated transcripts (5, 29–31). These findings suggest that NAT10 may preferentially reinforce transcripts associated with stress adaptation and malignant fitness. However, whether this apparent selectivity reflects direct substrate recognition by NAT10, recruitment by partner proteins, altered RNA accessibility under stress, or secondary consequences of transcriptional remodeling remains unresolved. Therefore, future studies combining acRIP-seq, NAT10 CLIP-based binding maps, RNA structural profiling, and catalytic loss-of-function rescue experiments will be necessary to define the molecular rules governing NAT10 target specificity.

### 2.2 Regulation of NAT10 expression and stability

Although NAT10 possesses intrinsic acetyltransferase activity, its oncogenic function in cancer is largely determined by upstream regulatory inputs that control its expression, stability, and spatial organization. Multiple signaling pathways converge to modulate NAT10 abundance and activity, forming an integrated upstream regulatory layer that links environmental stress, mechanical cues, metabolic state, and proteostasis to epitranscriptomic remodeling.

At the transcriptional level, the Hippo pathway effector Yes-associated protein 1 (YAP1) has emerged as a key activator of NAT10 expression (32). In several cancer contexts, YAP1 directly binds to the NAT10 promoter and enhances its transcription, thereby coupling mechanical signaling and extracellular matrix stiffness to RNA acetylation programs. Through this axis, biomechanical stress can indirectly amplify ac4C-mediated stabilization of transcripts involved in proliferation and metabolism, establishing a bridge between tissue growth pathways and epitranscriptomic control. Hypoxic signaling further reinforces this regulatory circuit. As a hallmark of solid tumors, hypoxia enhances NAT10 expression and function, contributing to feed-forward loops with HIF-1α (33, 34). By stabilizing glycolytic and metabolic transcripts, NAT10 amplifies hypoxia-driven adaptation, converting transient oxygen deprivation signals into sustained metabolic reprogramming.

Environmental and inflammatory stimuli also feed into NAT10 regulation. In gastric cancer, Helicobacter pylori (H. pylori) infection induces NAT10 expression, leading to ac4C-dependent stabilization of oncogenic transcripts such as mouse double minute 2 homolog (MDM2) and subsequent suppression of tumor protein p53 (p53) activity (35). This example illustrates how chronic inflammation can hijack epitranscriptomic regulation to facilitate tumorigenesis. Beyond transcriptional control, NAT10 protein stability is dynamically modulated by post-translational mechanisms. Lysine 2-hydroxyisobutyrylation (Khib) enhances NAT10 stability by preventing ubiquitin-mediated degradation, suggesting that intracellular metabolic intermediates can directly influence NAT10 abundance (36). In parallel, interactions with deubiquitinases protect NAT10 from proteasomal turnover, amplifying its steady-state levels in cancer cells and sustaining ac4C deposition.

Finally, NAT10 activity is shaped not only by abundance but also by spatial organization. Emerging evidence indicates that NAT10 participates in liquid–liquid phase separation, forming nuclear condensates that concentrate RNA processing machinery. Within these condensates, NAT10 may locally enhance ac4C deposition and coordinate splicing or RNA maturation events in a stress-responsive manner. Such phase-separated assemblies provide a mechanism for rapid, reversible modulation of NAT10 activity, allowing cancer cells to dynamically reorganize RNA regulatory programs in response to environmental challenges. Collectively, these transcriptional, metabolic, post-translational, and spatial regulatory inputs position NAT10 as a stress-integrating node whose activity reflects the broader adaptive state of the tumor cell.

## 3 Epitranscriptomic control by NAT10: building a molecular network

If Section 2 defined how NAT10 is activated and regulated, this section addresses how NAT10 executes its downstream functions at the transcriptome level. Rather than modifying isolated RNAs, NAT10 reshapes interconnected regulatory circuits. Through ac4C deposition, NAT10 amplifies specific oncogenic programs, coordinates translational output, and interfaces with chromatin signaling pathways. These multilayered effects establish NAT10 as a molecular network integrator rather than a linear pathway component.

### 3.1 mRNA stability and translation control

A central function of NAT10-mediated ac4C deposition is the stabilization and amplification of oncogenic transcripts that sustain malignant progression. Unlike transient transcriptional activation, ac4C modification prolongs mRNA half-life and ensures persistent protein production, thereby reinforcing oncogenic signaling at the post-transcriptional level (37). This mechanism enables tumor cells to maintain elevated expression of key regulators even under therapeutic or microenvironmental stress. Through selective stabilization of transcripts encoding drug resistance transporters such as multidrug resistance protein 1 (MDR1, ABCB1) and breast cancer resistance protein (BCRP, ABCG2), chromatin and cytoskeletal regulators including high mobility group AT-hook 1 (HMGA1) and keratin 8 (KRT8), extracellular matrix components such as laminin subunit beta 3 (LAMB3), and mitotic regulators such as kinesin family member 23 (KIF23), NAT10 simultaneously reinforces diverse functional programs spanning cell-cycle progression, epithelial–mesenchymal transition (EMT), adhesion signaling, and chemotherapy resistance (30, 31, 38). These effects are not isolated; rather, they represent coordinated reinforcement of interconnected oncogenic circuits that collectively sustain malignant phenotypes.

Beyond transcript stabilization, NAT10 further enhances protein output through layered translational control. ac4C deposition on mRNA promotes ribosome engagement, while NAT10-mediated acetylation of tRNA improves translational efficiency and fidelity at a systems level. The EGFR signaling axis illustrates this dual mechanism: enhanced tRNA acetylation increases EGFR protein synthesis, sustaining receptor signaling and facilitating resistance to tyrosine kinase inhibitors even in the absence of new mutations (39). Notably, metabolic enzymes are also preferentially stabilized by NAT10, including G6PD within the pentose phosphate pathway and LDHA and PFKM within glycolysis (40–44). Through coordinated control of RNA stability and translational capacity, NAT10 operates at the interface between transcript abundance and proteome output, transforming epitranscriptomic modification into sustained oncogenic execution.

Collectively, NAT10-mediated mRNA and tRNA ac4C modifications regulate transcript stability, translation efficiency, and stress adaptation. In addition to these substrates, NAT10 also mediates ac4C modification on 18S rRNA, which is critical for ribosome biogenesis and translational fidelity. NAT10 catalyzes N4-acetylation of cytidine residues within the 18S rRNA, contributing to proper pre-18S rRNA processing and 40S ribosomal subunit assembly (45). Loss of NAT10 or perturbation of rRNA ac4C impairs ribosome maturation, reduces global translational efficiency, and sensitizes cells to stress, highlighting its essential role in maintaining basal protein synthesis. These findings indicate that rRNA ac4C is a fundamental mechanism through which NAT10 supports cellular homeostasis in both normal and cancer cells.

### 3.2 Crosstalk between ac4C and other RNA modifications

Epitranscriptomic regulation rarely operates in isolation; instead, RNA modifications frequently interact to fine-tune transcript fate and signaling robustness. Emerging evidence suggests that ac4C does not function independently but intersects functionally with other RNA marks, particularly m6A. ac4C deposition may influence m6A distribution or function by altering RNA secondary structure, modulating accessibility of methyltransferase or demethylase complexes, or stabilizing transcripts that are concurrently m6A-modified. In certain contexts, ac4C and m6A appear to exert layered stabilization effects, enhancing transcript persistence and translational competence beyond what either modification could achieve alone. Such cooperative regulation may increase the resilience of oncogenic transcript expression, ensuring sustained signaling output under metabolic stress or therapeutic pressure.

Beyond structural interplay, functional convergence between ac4C and m6A pathways is exemplified by regulatory axes involving m6A reader proteins. YTHDC1(YTH Domain Containing 1), a nuclear m6A reader implicated in RNA export and processing, has been linked to pathways influenced by NAT10 activity, suggesting that ac4C-mediated transcript stabilization may intersect with m6A-dependent RNA handling (46–49). Moreover, NAT10 participates in alternative splicing regulation through phase separation–dependent acetylation of splicing factors such as SRSF2, influencing exon inclusion events including those affecting YTHDF1 isoforms (50). This creates a hierarchical regulatory cascade in which NAT10 modulates splicing machinery, reshapes m6A reader composition, and ultimately alters translational control. Through such multilayered interactions, NAT10 integrates ac4C deposition with broader epitranscriptomic networks, reinforcing transcript stability, RNA processing, and signaling amplitude across multiple regulatory levels.

### 3.3 Chromatin-associated RNA and transcriptional coupling

Recent advances extend NAT10’s influence beyond classical RNA metabolism into chromatin-associated regulatory processes, revealing an unexpected interface between epitranscriptomic modification and transcriptional control. Evidence suggests that NAT10-mediated acetylation of chromatin-associated tRNAs can modulate transcriptional co-activator activity in a manner independent of canonical translation (28, 51–54). Rather than serving solely as adapters in protein synthesis, these modified RNAs appear to function as regulatory scaffolds that interact with chromatin-associated complexes, influencing transcriptional output at specific genomic loci. This finding expands the conceptual scope of ac4C biology, linking RNA acetylation directly to epigenetic regulation and gene expression control.

At the chromatin level, NAT10 activity has been associated with modulation of p300/CBP acetyltransferase function and histone acetylation states (55). By influencing RNA scaffolds within chromatin-associated complexes, NAT10 may enhance transcriptional activation at oncogenic loci, thereby reinforcing proliferative and metabolic programs. This establishes a bidirectional axis in which epitranscriptomic remodeling shapes chromatin accessibility, and transcriptional activation further amplifies downstream adaptive pathways (56). In this framework, NAT10 is not confined to post-transcriptional regulation but participates in coupling RNA modification with transcriptional reinforcement.

Importantly, several NAT10-regulated transcripts are embedded within positive feedback circuits involving major oncogenic transcription factors, elevating NAT10 from a downstream modifier to a central network amplifier. Within the Wnt/β-catenin pathway, NAT10-mediated stabilization of transcripts that potentiate Wnt signaling strengthens proliferative and stemness-associated programs, while activated β-catenin signaling can reciprocally enhance NAT10 expression or activity, forming a self-sustaining loop (57, 58). Similarly, under hypoxic conditions, NAT10 upregulation reinforces glycolytic transcript stability, intensifies metabolic flux, and supports HIF-1α signaling; enhanced glycolysis and redox adaptation further stabilize HIF-1α activity, locking tumor cells into a hypoxia-adapted state (59). Through such reciprocal reinforcement across transcriptional, metabolic, and signaling layers, NAT10 transitions from a passive executor of upstream cues to a systems-level amplifier capable of sustaining oncogenic signaling, metabolic plasticity, and long-term adaptive tumor states.

**Figure 1:**
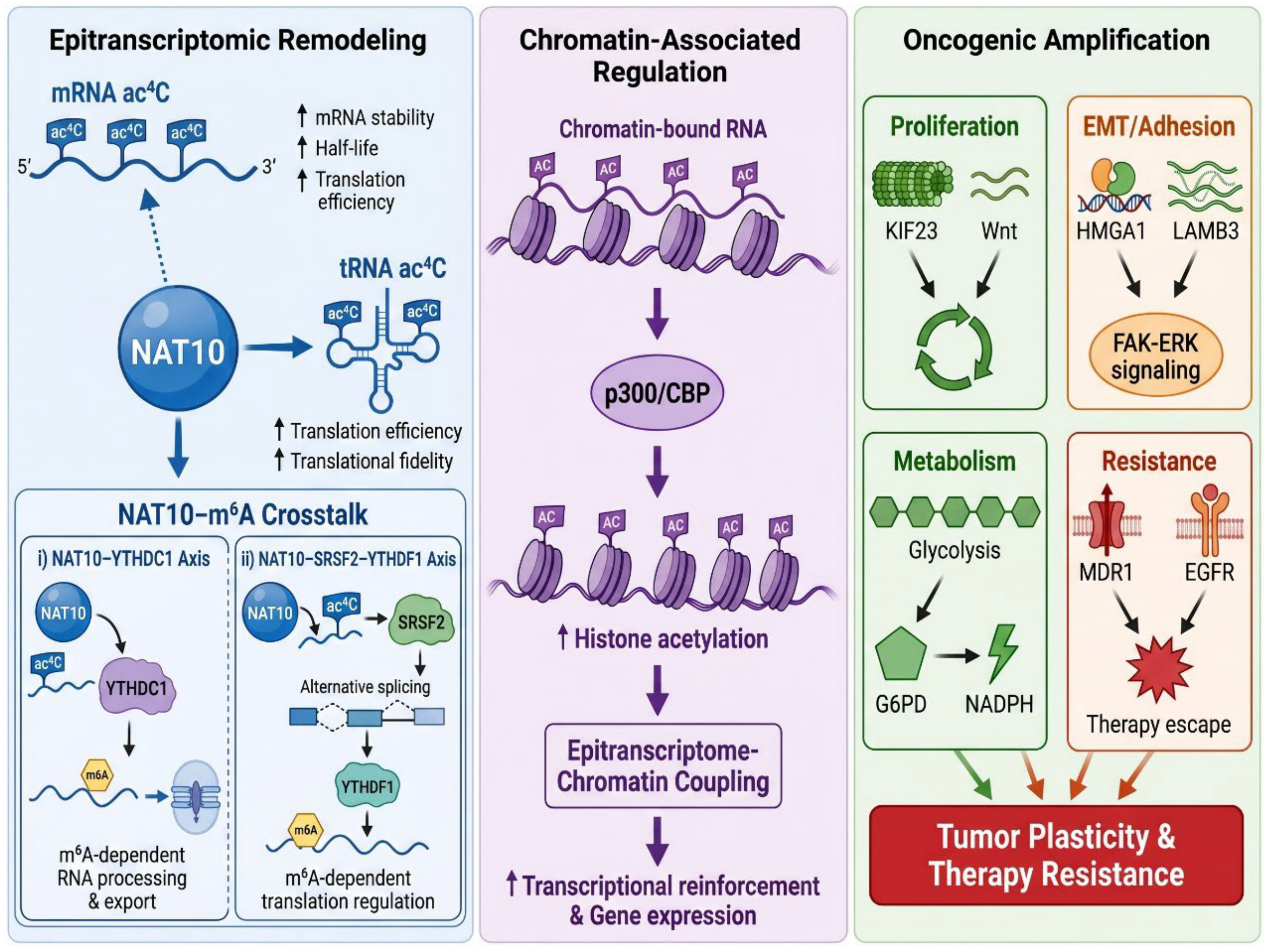
NAT10 as a central epitranscriptomic amplifier in cancer. NAT10-mediated ac4C deposition enhances mRNA stability and tRNA-dependent translation while intersecting with m6A regulation and alternative splicing. Beyond post-transcriptional control, NAT10 couples RNA acetylation to chromatin remodeling through p300/CBP-associated transcriptional activation. These coordinated mechanisms reinforce oncogenic signaling, metabolic rewiring, and therapeutic resistance. Feedback circuits involving β-catenin and HIF-1α further sustain NAT10 activity, promoting tumor plasticity and adaptive survival.

Taken together, NAT10-mediated ac4C modification exerts broad regulatory control across multiple layers of gene expression, encompassing transcript stability, translational efficiency, alternative splicing, chromatin-associated transcription, and oncogenic signaling feedback circuits (60). Rather than operating within a single linear pathway, NAT10 integrates interconnected molecular networks that bridge RNA metabolism, protein synthesis, chromatin remodeling, and signal transduction (61). As illustrated in **Figure 1**, NAT10 functions as a central epitranscriptomic hub that integrates RNA stabilization, translational amplification, and chromatin coupling to reinforce oncogenic signaling and tumor plasticity. Through this multilayered coordination, NAT10 functions as a molecular amplifier of tumor plasticity—stabilizing oncogenic transcripts, enhancing metabolic flux, and reinforcing adaptive signaling programs that collectively sustain malignant progression and therapeutic resistance. A comprehensive summary of NAT10-regulated transcripts, their RNA substrates, and functional outputs across cancer types is provided in **Table 1**.

**Table 1:** NAT10-regulated targets and functional outputs in cancer.

Target/axis	RNA substrate	Functional output	Cancer context	Biological consequence
MDR1 (ABCB1), BCRP (ABCG2)	mRNA	Transcript stabilization	Breast, bladder	Drug efflux, chemoresistance
HMGA1, KRT8	mRNA	Increased stability	Prostate	EMT, metastasis
LAMB3	mRNA	ECM signaling reinforcement	PDAC	FAK/ERK activation, adhesion
KIF23	mRNA	Cell-cycle progression	CRC	Wnt/β-catenin activation
EGFR	tRNA-mediated translation	Enhanced protein synthesis	Multiple	TKI resistance
G6PD	mRNA	PPP activation	Hepatoblastoma	NADPH production, redox balance
LDHA, PFKM	mRNA	Glycolytic flux	TNBC, cervical	Lactate accumulation, immune suppression
ATP6V0E1	mRNA	Lysosomal acidification	ESCC	Metastasis
DDR-related genes	mRNA	DNA repair reinforcement	Bladder	Cisplatin resistance
FOXP1/JunB	mRNA	Metabolic–immune coupling	Cervical, TNBC	Glycolysis + immune suppression

## 4 NAT10-driven metabolic reprogramming: an engine of tumor plasticity

Metabolic rewiring is a defining hallmark of cancer and a critical determinant of tumor adaptability. Increasing evidence places NAT10 at the center of this metabolic transformation. Through ac4C-mediated stabilization of metabolic transcripts and integration with oncogenic signaling pathways, NAT10 establishes self-reinforcing metabolic circuits that sustain biosynthetic demand, redox balance, and stress adaptation (62, 63). Rather than passively responding to metabolic cues, NAT10 actively amplifies metabolic plasticity, enabling tumor cells to dynamically shift between metabolic states in response to hypoxia, nutrient limitation, and therapeutic pressure.

### 4.1 Glycolysis addiction: reinforcing hypoxic metabolic circuits

One of the most consistent metabolic outputs of NAT10 activation is enhanced glycolytic flux. Multiple studies demonstrate that NAT10 stabilizes transcripts encoding key glycolytic enzymes, including LDHA and PFKM, thereby strengthening rate-limiting steps in the glycolytic pathway (64). This stabilization promotes sustained lactate production and ATP generation, supporting rapid proliferation and biomass accumulation. Importantly, NAT10 is integrated into a feedback loop with HIF-1α, the master regulator of hypoxia-responsive metabolism. Hypoxic signaling can induce NAT10 expression, while NAT10-mediated stabilization of glycolytic transcripts reinforces HIF-1α activity through enhanced metabolic flux. This bidirectional interaction forms a self-amplifying circuit that locks tumor cells into a glycolysis-dependent state—commonly referred to as “glycolytic addiction.” Additional regulatory axes further expand this network. NAT10-dependent stabilization of transcriptional regulators such as FOXP1 and JunB contributes to glycolytic gene activation and immunosuppressive metabolic remodeling (65, 66). Through these mechanisms, NAT10 not only fuels intrinsic metabolic demands but also shapes the tumor microenvironment by promoting lactate-driven immune suppression. Thus, NAT10-driven glycolysis extends beyond energy production to encompass metabolic–immune coupling.

### 4.2 Serine and amino acid metabolism: supporting biosynthesis and stemness

Beyond glycolysis, NAT10 plays a critical role in amino acid metabolism, particularly in contexts requiring enhanced biosynthetic capacity. In acute myeloid leukemia (AML), NAT10-mediated ac4C reprograms serine metabolism, promoting nucleotide synthesis and redox homeostasis that support leukemic stem cell maintenance (67). By stabilizing transcripts involved in serine biosynthesis pathways, NAT10 sustains anabolic flux required for rapid proliferation and stemness preservation. A parallel regulatory module involves the ATF4–ASNS axis, which governs adaptive amino acid responses. NAT10 enhances ATF4 signaling and stabilizes ASNS transcripts, promoting asparagine biosynthesis under metabolic stress (68). Elevated asparagine levels not only support protein synthesis but also buffer against nutrient deprivation, enhancing tumor survival in hostile microenvironments. Through these mechanisms, NAT10 coordinates amino acid metabolism with cellular stress responses, reinforcing tumor resilience.

### 4.3 Pentose phosphate pathway activation: redox and anabolic control

The pentose phosphate pathway (PPP) represents another key metabolic node influenced by NAT10 (69). The YAP1–NAT10–G6PD axis exemplifies how oncogenic transcriptional programs intersect with epitranscriptomic regulation. YAP1 transcriptionally upregulates NAT10, which in turn stabilizes G6PD mRNA via ac4C modification. Enhanced G6PD expression increases NADPH production, supporting reductive biosynthesis and protection against oxidative stress (70). This axis forms a feed-forward circuit: YAP1 promotes NAT10, NAT10 reinforces PPP flux, and increased NADPH availability supports further proliferation and survival. Through PPP activation, NAT10 ensures sufficient nucleotide production for DNA replication while simultaneously maintaining antioxidant capacity—both essential for sustaining rapid tumor growth (71, 72).

### 4.4 Organelle adaptation: lysosomal acidification and metabolic recycling

Metabolic plasticity also depends on organelle-level adaptation. Emerging evidence indicates that NAT10 regulates lysosomal function by stabilizing ATP6V0E1 mRNA, a subunit of the vacuolar ATPase complex responsible for lysosomal acidification (73). Enhanced lysosomal acidification facilitates macromolecule degradation and nutrient recycling, supporting anabolic metabolism and metastatic competence. By influencing lysosomal activity, NAT10 links RNA acetylation to autophagic flux and intracellular nutrient availability. This mechanism enables tumor cells to maintain metabolic flexibility during nutrient deprivation or therapeutic stress.

**Figure 2:**
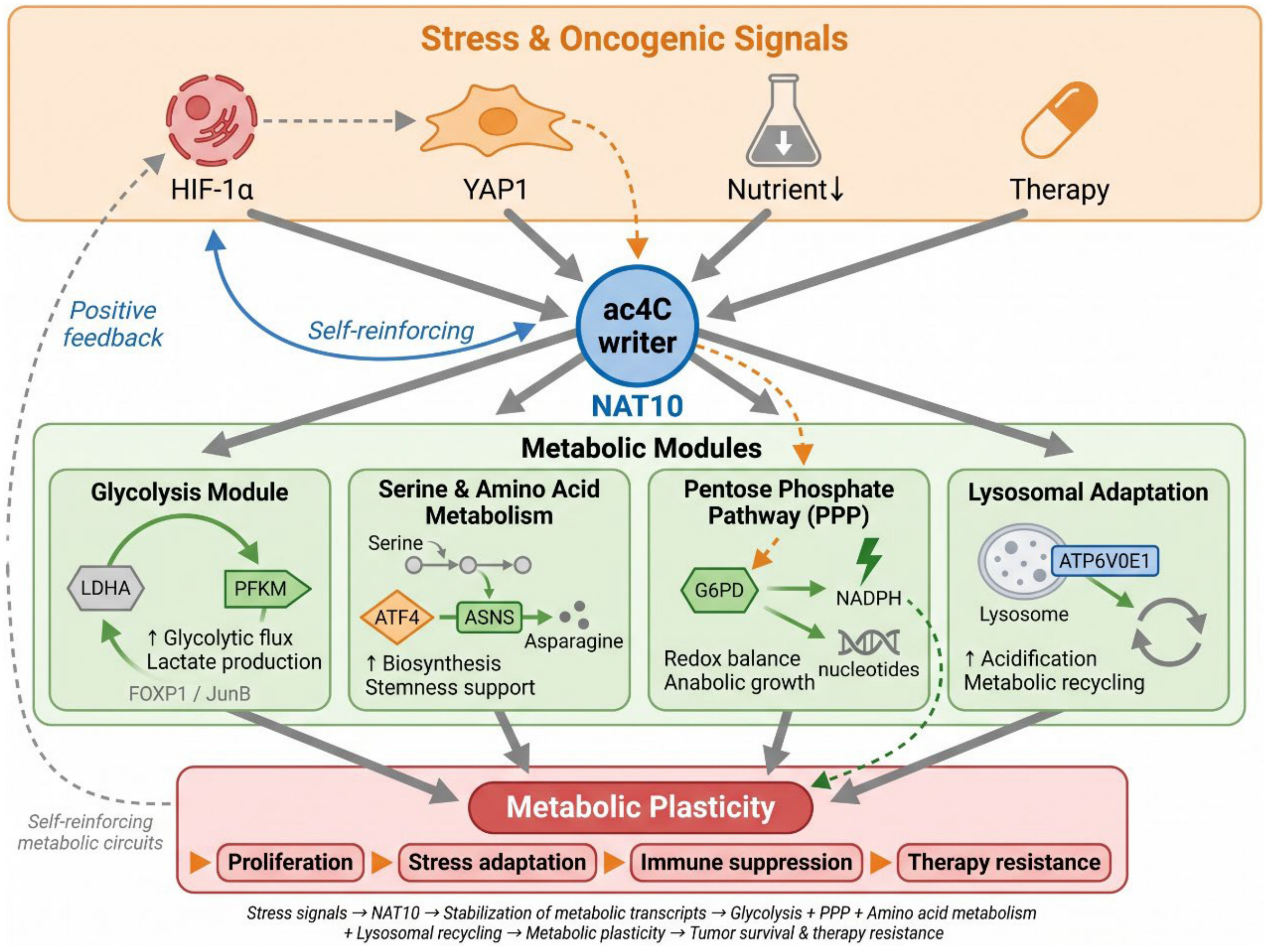
NAT10-driven metabolic reprogramming in cancer. Upstream stress signals activate NAT10, which stabilizes metabolic transcripts via ac4C deposition. NAT10 enhances glycolysis (LDHA, PFKM), serine and amino acid metabolism (ATF4–ASNS axis), pentose phosphate pathway flux (G6PD–NADPH), and lysosomal acidification (ATP6V0E1). These interconnected pathways form self-reinforcing circuits with HIF-1α and YAP1, promoting metabolic plasticity, redox balance, anabolic growth, immune suppression, and therapy resistance.

Collectively, NAT10 coordinates glycolysis, amino acid biosynthesis, pentose phosphate pathway activation, and organelle-mediated nutrient recycling (74–76). These pathways are not independently regulated; instead, they form interconnected amplification circuits. Glycolysis supports PPP flux, PPP-derived NADPH maintains redox balance, amino acid metabolism sustains protein synthesis, and lysosomal acidification enables metabolic recycling. By stabilizing transcripts at critical metabolic checkpoints, NAT10 transforms transient metabolic responses into sustained adaptive states. This capacity to reinforce multiple metabolic modules simultaneously explains how NAT10 drives tumor plasticity across diverse cancer types. Rather than acting as a simple metabolic regulator, NAT10 functions as an epitranscriptomic conductor orchestrating metabolic flexibility, enabling cancer cells to transition between proliferative, invasive, and therapy-resistant phenotypes. As illustrated in **Figure 2**, NAT10 integrates stress signaling with ac4C-mediated stabilization of metabolic transcripts to establish self-reinforcing circuits that drive metabolic plasticity and tumor adaptation.

## 5 NAT10 and tumor microenvironment remodeling: the ecosystem layer

Although NAT10 is frequently discussed in the context of cancer-cell-intrinsic adaptation, it also has important physiological functions in normal cells. NAT10 participates in rRNA processing, tRNA acetylation, ribosome biogenesis, translational fidelity, DNA damage responses, centrosome regulation, nuclear architecture, and stress adaptation (45, 77, 78). These basal functions indicate that NAT10 is not a tumor-specific factor, and therefore therapeutic strategies targeting NAT10 must carefully consider potential toxicity in normal proliferative tissues and immune compartments. Recent evidence also indicates that NAT10 can regulate antigen-stimulated T-cell activation and proliferation, further supporting the need to evaluate immune-cell consequences of systemic NAT10 inhibition (79). Tumor cells may exhibit increased dependence on NAT10 under hypoxia, metabolic stress, and therapy-induced pressure, but this therapeutic window remains to be clearly defined. At present, direct evidence that NAT10 regulates tumor-infiltrating immune cells in a cell-autonomous manner remains limited. Most available studies suggest that NAT10 influences antitumor immunity primarily through cancer-cell-intrinsic or tumor-microenvironmental mechanisms, including glycolytic reprogramming, lactate accumulation, PD-L1-associated immune suppression, and reduced cytotoxic CD8^+^ T-cell activity (53, 80, 81). Whether NAT10 directly controls the metabolism, exhaustion, cytokine production, or effector function of T cells, macrophages, dendritic cells, or myeloid-derived suppressor cells within tumors remains an open question. Of note, NAT10 has been implicated in macrophage activation in inflammatory disease models, suggesting that immune-cell-intrinsic NAT10 functions are biologically plausible but remain insufficiently characterized in the tumor-infiltrating immune compartment (82). Immune-cell-specific Nat10 knockout models, single-cell ac4C mapping, and spatial immune profiling will be required to clarify these direct immune-cell-autonomous functions. While NAT10-driven metabolic reprogramming supports intrinsic tumor growth, its influence extends beyond cancer cells to reshape the surrounding microenvironment. Increasing evidence indicates that NAT10-mediated ac4C remodeling contributes to immune suppression, vascular dysfunction, and metastatic niche formation. Through coordinated regulation of immune modulators, adhesion molecules, and metabolic factors, NAT10 helps construct a tumor ecosystem that favors immune evasion, invasion, and organ colonization. Thus, NAT10 functions not only as an intracellular regulator but also as an architect of the tumor microenvironment (TME).

### 5.1 Immune suppression and CD8^+^ T-cell dysfunction

One of the most consequential outcomes of NAT10 activation is the establishment of an immunosuppressive microenvironment. Multiple studies demonstrate that NAT10-driven ac4C modification stabilizes transcripts involved in metabolic reprogramming and inflammatory signaling, indirectly impairing cytotoxic T-cell function. NAT10 activity has been associated with an “immune desert” phenotype characterized by reduced CD8^+^ T-cell infiltration and diminished effector function (83). Mechanistically, enhanced glycolysis and lactate production—driven by NAT10-stabilized glycolytic enzymes—can create a metabolically hostile environment for T cells, limiting their cytotoxic capacity. In parallel, NAT10-dependent signaling pathways intersect with PD-1/PD-L1 axes, reinforcing checkpoint-mediated immune suppression and reducing responsiveness to immunotherapy (80, 84–87). Beyond T cells, NAT10 also influences myeloid cell polarization. Stabilization of transcripts that promote pro-tumorigenic cytokine signaling favors the accumulation and polarization of M2-like macrophages and other immunosuppressive myeloid populations. These cells further suppress cytotoxic immunity and secrete growth factors that enhance tumor progression. Through metabolic–immune coupling, NAT10 integrates intrinsic oncogenic signaling with extrinsic immune remodeling.

### 5.2 Vascular abnormalization and immune exclusion

Vascular remodeling represents another critical dimension of NAT10-mediated microenvironmental control (88–90). The NAT10/XIST/YAP1/VEGFA axis exemplifies how epitranscriptomic regulation intersects with angiogenic signaling. YAP1-mediated transcriptional activation of NAT10, together with NAT10-dependent RNA stabilization events, enhances VEGFA signaling and contributes to aberrant vascular architecture. Abnormal tumor vasculature is characterized by disorganized, leaky vessels and impaired perfusion, leading to hypoxia and immune exclusion (91–94). By promoting VEGFA-driven angiogenesis, NAT10 indirectly reinforces hypoxic conditions that further sustain its own metabolic feedback circuits. This creates a self-reinforcing loop in which vascular dysfunction amplifies hypoxia, hypoxia enhances NAT10 activity, and NAT10 further destabilizes vascular integrity. Importantly, vascular abnormalization restricts immune cell infiltration, compounding the immune desert phenotype (95). Thus, NAT10-mediated angiogenic signaling not only supports nutrient supply but also establishes physical and metabolic barriers to antitumor immunity.

### 5.3 Metastatic niche formation and organ colonization

**Figure 3:**
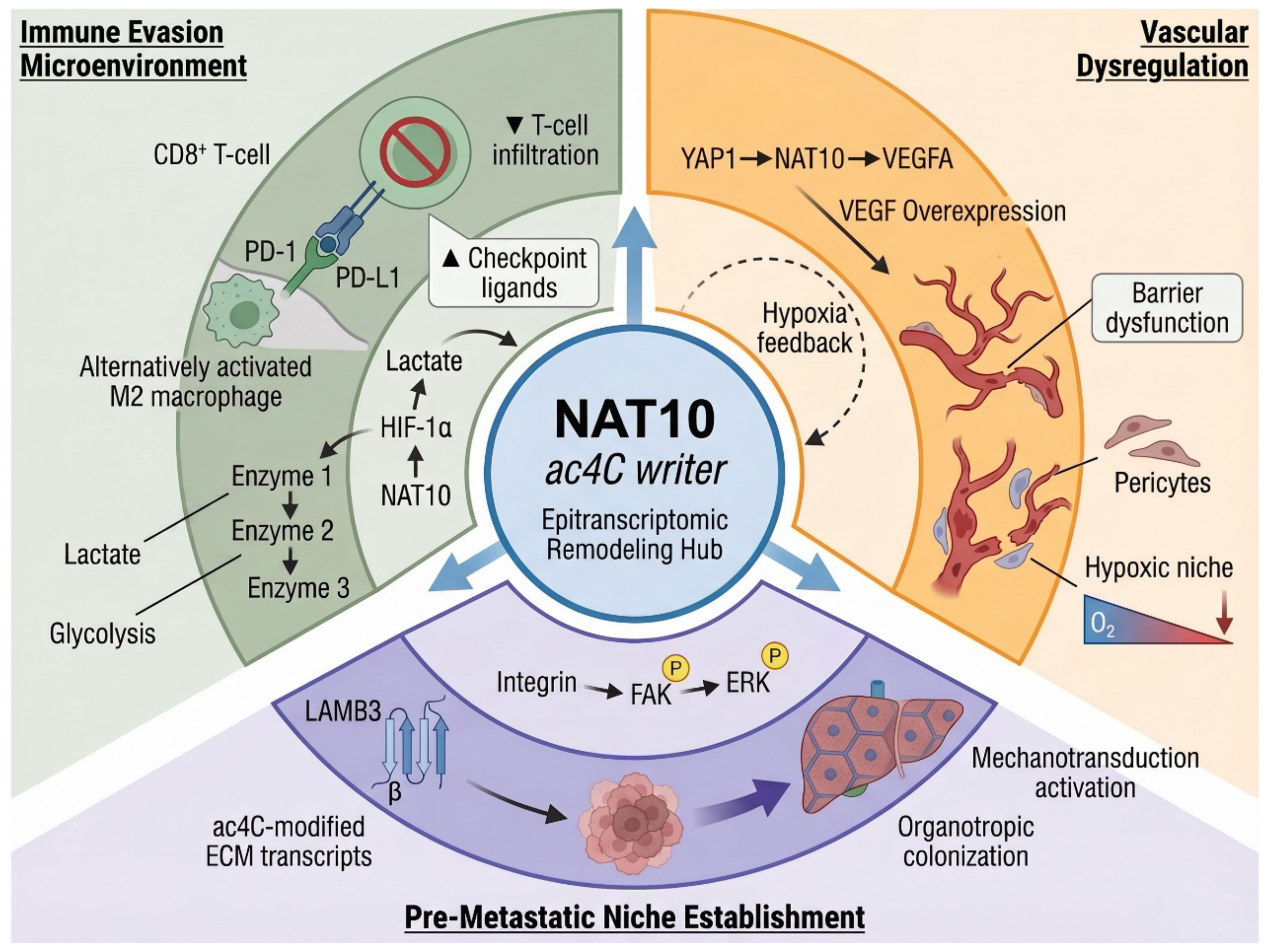
NAT10-driven tumor microenvironment remodeling. NAT10-mediated ac4C remodeling promotes immune suppression, vascular abnormalization, and metastatic niche formation. By enhancing glycolysis and lactate production, NAT10 impairs CD8^+^ T-cell function and promotes M2 macrophage polarization. Concurrently, the YAP1–NAT10–VEGFA axis drives aberrant angiogenesis and hypoxia, reinforcing immune exclusion. Stabilization of adhesion and ECM-related transcripts further facilitates organ colonization. These interconnected processes establish a permissive tumor ecosystem that supports immune evasion and therapeutic resistance.

Metastasis requires coordinated interactions between tumor cells and distant microenvironments. NAT10 contributes to this process by stabilizing transcripts involved in adhesion signaling, extracellular matrix (ECM) remodeling, and immune modulation. NAT10-dependent polarization of M2 macrophages promotes a pro-metastatic niche characterized by immunosuppression, matrix remodeling, and growth factor secretion. In gastric cancer models, NAT10 activity has been linked to enhanced liver colonization, partly through regulation of adhesion molecules that facilitate tumor cell attachment to hepatic tissue (96). At the molecular level, stabilization of ECM-related transcripts such as LAMB3 activates integrin–FAK–ERK signaling pathways, strengthening cell–matrix interactions and promoting survival in foreign microenvironments (97). These adhesion-mediated signals not only facilitate metastatic seeding but also activate downstream proliferative and anti-apoptotic pathways necessary for outgrowth. Through combined effects on macrophage polarization, vascular remodeling, and adhesion signaling, NAT10 orchestrates the formation of permissive metastatic niches. Rather than acting solely within tumor cells, NAT10 shapes the structural and immunological landscape that enables metastatic dissemination. As illustrated in **Figure 3**, NAT10 functions as a central architect of tumor ecosystem remodeling by integrating immune suppression, vascular dysfunction, and metastatic niche formation.

## 6 NAT10 in therapy resistance: a convergence node of adaptive escape

**Figure 4:**
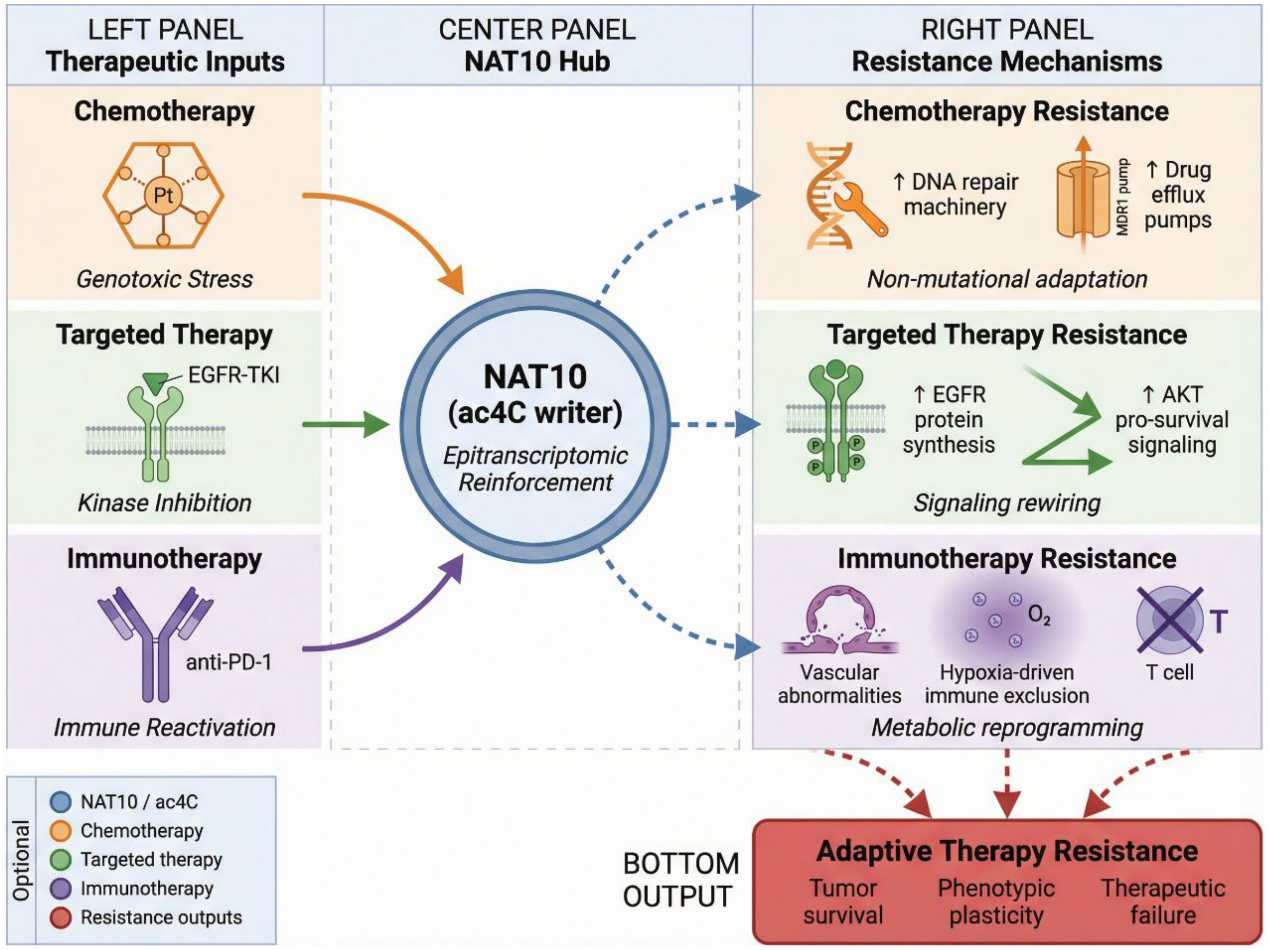
NAT10 as a convergence node in therapy resistance. NAT10-mediated ac4C stabilization reinforces multiple adaptive resistance mechanisms. By enhancing DNA repair and drug efflux, NAT10 promotes chemoresistance; by increasing EGFR translation and AKT survival signaling, it sustains resistance to targeted therapies; and by driving vascular dysfunction, metabolic immunosuppression, and immune exclusion, it contributes to immune checkpoint blockade failure. These interconnected pathways converge to establish multilayered, non-mutational adaptive therapy resistance.

Therapy resistance remains one of the most formidable challenges in oncology, arising not only from genetic mutations but also from dynamic, non-genetic adaptive programs. Emerging evidence positions NAT10-mediated ac4C modification as a central regulator of these adaptive states. By stabilizing transcripts involved in DNA repair, drug transport, receptor signaling, metabolism, and immune modulation, NAT10 reinforces multiple resistance mechanisms simultaneously (98–100). Rather than conferring resistance through a single pathway, NAT10 establishes a multilayered defense architecture that enables tumor cells to withstand chemotherapy, targeted therapy, and immunotherapy. As illustrated in **Figure 4**, NAT10 integrates chemotherapy, targeted therapy, and immunotherapy resistance through coordinated ac4C-dependent reinforcement of adaptive signaling networks.

### 6.1 Chemotherapy resistance

Resistance to cytotoxic chemotherapy frequently emerges through adaptive enhancement of cellular defense mechanisms rather than solely through permanent genetic alterations. In the case of cisplatin, which induces DNA crosslinks and replication stress, tumor cells often survive by upregulating DNA damage repair (DDR) pathways (101). NAT10 contributes to this adaptive response by stabilizing transcripts encoding key components of the DNA repair machinery through ac4C modification. In bladder cancer models, NAT10-mediated RNA stabilization enhances repair efficiency of cisplatin-induced lesions, reduces DNA damage accumulation, and attenuates apoptosis (102). By reinforcing DDR at the post-transcriptional level, NAT10 transforms transient repair responses into sustained protective programs. This highlights a broader principle: epitranscriptomic stabilization can amplify canonical stress-response pathways, providing a non-genetic route to chemoresistance that operates independently of mutation acquisition.

In parallel, NAT10 strengthens chemotherapy resistance through regulation of drug efflux mechanisms. ac4C deposition on transcripts encoding ATP-binding cassette (ABC) transporters, including MDR1 (ABCB1) and BCRP (ABCG2), increases their stability and expression. Elevated transporter levels enhance the export of chemotherapeutic agents, reducing intracellular drug concentrations and diminishing cytotoxic efficacy. By simultaneously augmenting DNA repair capacity and sustaining drug efflux, NAT10 establishes a dual-layered resistance architecture (103–105). Notably, pharmacologic inhibition of NAT10 has been shown to decrease transporter expression and partially restore chemosensitivity, underscoring its translational potential. Together, these findings position NAT10 as a central mediator of non-mutational chemotherapy resistance, integrating repair reinforcement and drug export into a coordinated adaptive program.

### 6.2 Targeted therapy resistance

Resistance to targeted therapies directed against receptor tyrosine kinases (RTKs) frequently arises through adaptive reinforcement of signaling networks rather than solely through secondary mutations (106). In this context, NAT10 represents an epitranscriptomic mechanism that sustains oncogenic signaling amplitude even under pharmacologic inhibition. By enhancing EGFR protein synthesis through tRNA-mediated translational control, NAT10 increases receptor abundance and signaling output despite the presence of tyrosine kinase inhibitors (TKIs) (107). Importantly, this mechanism operates independently of new genetic alterations, illustrating how RNA-level regulation can bypass mutation-centric models of resistance. Through increased translational efficiency, NAT10 effectively compensates for partial receptor inhibition, maintaining downstream signaling flux and promoting therapeutic escape.

Beyond receptor-level amplification, NAT10 also reinforces downstream survival pathways. Stabilization of transcripts involved in AKT signaling enhances prosurvival and anti-apoptotic responses under therapeutic stress. Elevated AKT activity not only promotes cell survival but also supports metabolic adaptation, enabling tumor cells to tolerate signaling disruption (108). By simultaneously amplifying upstream receptor expression and downstream survival effectors, NAT10 establishes a multilayered buffering system that attenuates the impact of targeted inhibition. This coordinated reinforcement transforms partial pathway blockade into incomplete therapeutic suppression, underscoring NAT10’s role as a central mediator of non-mutational resistance to targeted therapies.

### 6.3 Immune checkpoint resistance

Resistance to immune checkpoint blockade (ICB) often arises not from intrinsic defects in antigen presentation or T-cell recognition, but from microenvironmental barriers that prevent effective immune engagement (109–111). Increasing evidence suggests that NAT10 contributes to this form of resistance by orchestrating coordinated changes in vascular architecture, metabolic state, and immunosuppressive signaling. Rather than acting directly on immune checkpoint genes alone, NAT10 reshapes the tumor ecosystem in ways that collectively undermine antitumor immunity.

One major axis involves aberrant vascular remodeling mediated through the NAT10/XIST/YAP1/VEGFA pathway. By promoting VEGFA-driven angiogenesis and vascular dysfunction, NAT10 contributes to the formation of disorganized, poorly perfused tumor vasculature. Such abnormal vessels generate hypoxic niches and create structural barriers that limit CD8^+^ T-cell infiltration (112). The resulting “immune-excluded” phenotype reduces the accessibility of effector T cells to tumor parenchyma, thereby diminishing responsiveness to PD-1/PD-L1 blockade. In this setting, immune checkpoint inhibition fails not because T cells cannot recognize tumor antigens, but because they are physically and metabolically restricted from reaching their targets.

Concurrently, NAT10-associated metabolic and vascular programs may contribute to immunosuppressive TME states that limit effective immune checkpoint blockade. However, these links remain largely preclinical and require validation in immunocompetent tumor models. Through this integrated remodeling of tumor ecology, NAT10 functions as an upstream regulator of immune checkpoint resistance, linking epitranscriptomic control to ecosystem-level therapeutic failure. The diverse mechanisms by which NAT10 contributes to metabolic adaptation, microenvironmental remodeling, and therapy resistance are systematically summarized in **Table 2**.

**Table 2:** NAT10 in tumor ecosystem remodeling and therapy resistance.

Adaptive layer	NAT10-driven mechanism	Downstream effect	Therapeutic implication
Metabolic rewiring	ac4C stabilization of glycolytic & PPP enzymes	Glycolysis addiction, redox resilience	Metabolic vulnerability
DNA repair	Stabilization of DDR transcripts	Chemotherapy resistance	Combine with genotoxic drugs
Drug efflux	MDR1/BCRP upregulation	Reduced intracellular drug concentration	Sensitize to chemotherapy
RTK signaling	tRNA-mediated EGFR translation	Targeted therapy escape	Combine with TKIs
AKT signaling	Survival transcript stabilization	Apoptosis resistance	Combine with PI3K/AKT inhibitors
Angiogenesis	NAT10/XIST/YAP1/VEGFA axis	Vascular abnormalization	Combine with anti-angiogenics
Immune suppression	Lactate + M2 polarization	Immune exclusion	Combine with PD-1 blockade
Metastatic niche	ECM–FAK–ERK signaling	Liver colonization	Anti-adhesion strategies

## 7 Therapeutic targeting of NAT10: from mechanistic insight to translational opportunity

**Figure 5:**
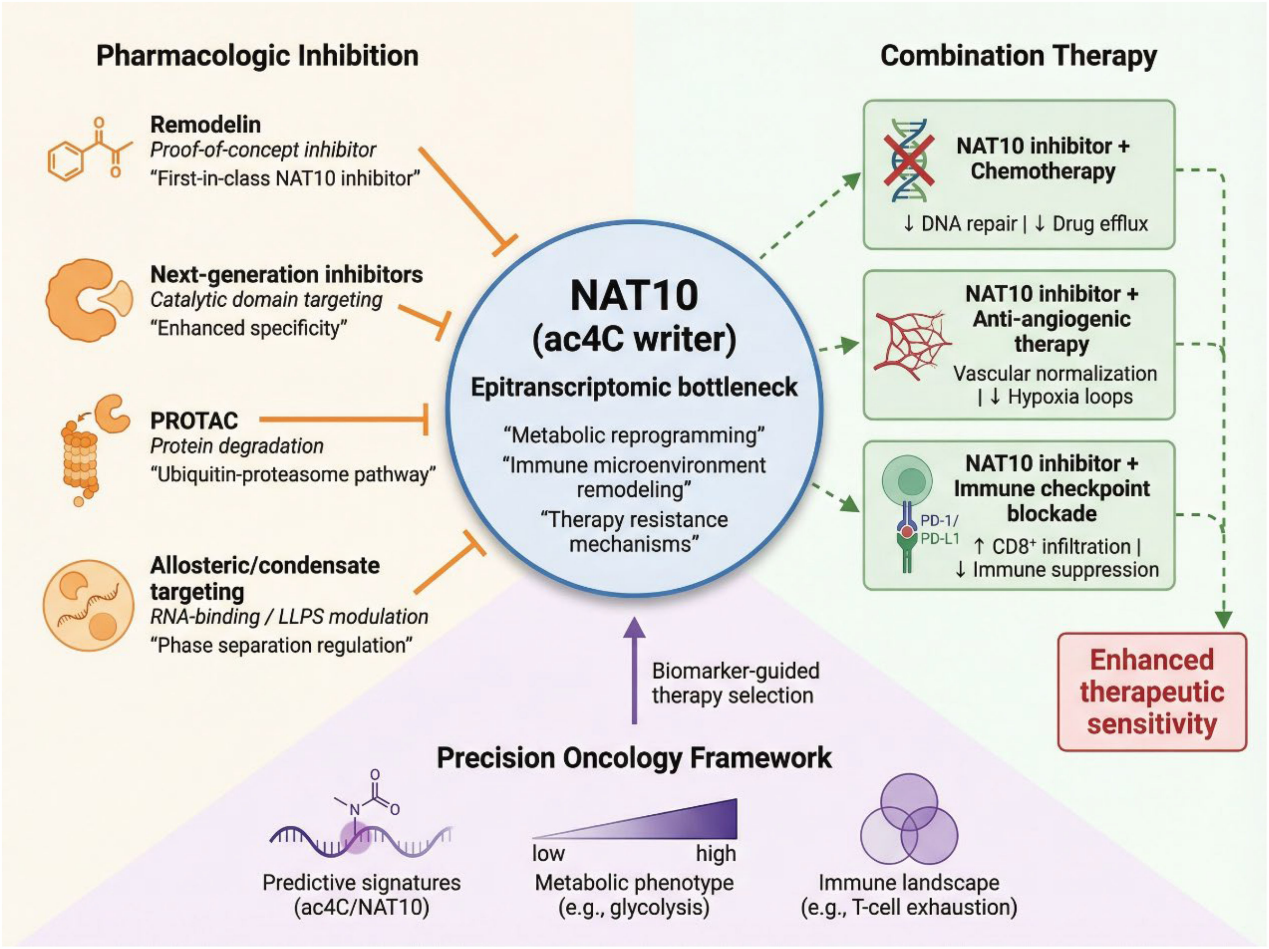
Therapeutic targeting of NAT10 in cancer. NAT10 represents an epitranscriptomic bottleneck integrating metabolic rewiring, immune remodeling, and therapy resistance. Pharmacologic strategies—including catalytic inhibitors, PROTAC-mediated degradation, and condensate modulation—aim to suppress NAT10 activity. Combination approaches with chemotherapy, anti-angiogenic agents, and immune checkpoint blockade may enhance therapeutic sensitivity. Integration of NAT10 expression and ac4C-related signatures provides a framework for biomarker-guided patient stratification and precision oncology.

Given its central role in metabolic rewiring, immune remodeling, and therapy resistance, NAT10 represents an attractive therapeutic target. Unlike many downstream oncogenic effectors, NAT10 occupies a regulatory bottleneck position, integrating multiple adaptive pathways at the RNA level. Targeting NAT10 therefore offers the potential to disrupt resistance programs simultaneously across metabolic, proliferative, and immunological axes. This section outlines current pharmacological strategies, rational combination approaches, and emerging biomarker frameworks for clinical translation. As illustrated in **Figure 5**, targeting NAT10 offers a strategy to destabilize adaptive resistance networks through pharmacologic inhibition, rational combination therapy, and biomarker-guided stratification. Importantly, therapeutic targeting of NAT10 remains at a preclinical stage. To date, no NAT10 inhibitor has entered clinical trials, and currently available compounds have mainly been used as experimental tools in cellular, organoid, xenograft, or patient-derived xenograft models. Therefore, the therapeutic strategies discussed below should be interpreted as preclinical hypotheses rather than clinically validated or trial-ready interventions. In particular, rational combinations involving NAT10 inhibition and chemotherapy, targeted therapy, anti-angiogenic therapy, or immune checkpoint blockade require rigorous pharmacological optimization, toxicity assessment, biomarker development, and validation in immunocompetent and clinically relevant models before clinical translation can be considered.

### 7.1 Pharmacological inhibition of NAT10

Pharmacological targeting of NAT10 has emerged as a promising translational strategy, motivated by its central role in epitranscriptomic regulation, metabolic adaptation, and therapy resistance. Among currently available compounds, Remodelin represents the earliest proof-of-concept inhibitor of NAT10. Initially identified in studies of nuclear architecture and laminopathies, Remodelin was later found to inhibit NAT10 acetyltransferase activity, thereby reducing ac4C deposition in cellular models (113–117). Although not originally developed for oncology, preclinical studies suggest that Remodelin-mediated NAT10 inhibition can suppress tumor growth, attenuate oncogenic signaling, and partially reverse chemoresistance phenotypes. These findings provide compelling evidence that NAT10 is pharmacologically targetable and that its enzymatic activity is functionally relevant in cancer. However, Remodelin is not an ideal therapeutic candidate. Its incomplete specificity, moderate potency, and suboptimal pharmacokinetic properties limit its translational potential. Moreover, as a first-generation inhibitor, Remodelin does not fully address questions of selectivity within the broader acetyltransferase family. These limitations highlight the urgent need for next-generation compounds with improved biochemical selectivity, enhanced cellular potency, and favorable drug-like characteristics.

Recent advances in structural modeling, virtual screening, and high-throughput compound libraries have begun to facilitate the identification of additional small molecules capable of inhibiting NAT10 catalytic activity. Rational drug design targeting the conserved acetyltransferase domain offers a promising path forward. Structural insights into the acetyl-CoA binding pocket and substrate recognition interfaces may enable the development of highly selective inhibitors that directly block ac4C deposition with minimal off-target effects. Beyond catalytic inhibition, alternative targeting strategies are conceptually attractive and may provide broader suppression of NAT10-driven adaptive programs. Proteolysis-targeting chimeras (PROTACs) designed to degrade NAT10 protein could reduce overall enzyme abundance rather than merely inhibiting its activity (118–122). Allosteric inhibitors that disrupt RNA binding or protein–protein interactions may interfere with substrate recognition or regulatory complex formation. Additionally, given NAT10’s involvement in phase-separated condensates, pharmacologic modulation of condensate formation represents an emerging and innovative approach. Considering NAT10’s integration into proteostasis and signaling networks, strategies that reduce total protein levels may achieve more comprehensive disruption of oncogenic circuits than catalytic inhibition alone.

### 7.2 Rational combination strategies

Given that NAT10 functions as an upstream integrator of multiple adaptive pathways—including DNA repair, metabolic rewiring, angiogenesis, and immune suppression—its inhibition is unlikely to achieve maximal efficacy as monotherapy in advanced malignancies. Instead, NAT10 targeting is conceptually best positioned as a sensitizing strategy that destabilizes adaptive networks and enhances responsiveness to established therapies. By disrupting epitranscriptomic reinforcement of survival programs, NAT10 inhibition may convert reversible adaptive states into therapeutically exploitable vulnerabilities. One of the most direct applications lies in combination with chemotherapy. NAT10 contributes to chemoresistance by stabilizing transcripts involved in DNA damage repair and drug efflux transport. Inhibiting NAT10 has the potential to simultaneously reduce repair capacity and diminish intracellular drug export, thereby amplifying genotoxic stress induced by agents such as cisplatin. Rather than merely increasing DNA damage, this strategy aims to prevent tumor cells from mounting effective compensatory responses, transforming transient injury into sustained cytotoxicity. By targeting resistance at the regulatory root, NAT10 inhibition may lower the threshold for chemotherapy efficacy.

A parallel rationale supports combination with anti-angiogenic therapy. Through the NAT10/XIST/YAP1/VEGFA axis, NAT10 promotes aberrant angiogenesis and hypoxia-driven metabolic adaptation (123–126). Combining NAT10 inhibition with anti-angiogenic agents may normalize tumor vasculature, alleviate hypoxia-induced feedback loops, and enhance therapeutic delivery. Importantly, suppressing NAT10 could dampen hypoxia-dependent glycolytic amplification circuits, disrupting the metabolic–vascular crosstalk that sustains tumor adaptation. This dual targeting of vascular structure and metabolic plasticity may produce synergistic antitumor effects. Perhaps most compelling is the integration of NAT10 inhibition with immune checkpoint blockade. NAT10-driven glycolysis, lactate accumulation, and vascular dysfunction contribute to immune exclusion and reduced CD8^+^ T-cell activity. By attenuating these metabolic and structural barriers, NAT10 inhibition may convert immunologically “cold” tumors into more permissive microenvironments. Preclinical findings suggest that suppression of NAT10 can reduce immunosuppressive signaling and improve T-cell infiltration, thereby enhancing responsiveness to PD-1/PD-L1 blockade (127). In this context, NAT10 inhibition functions as a microenvironmental reprogramming strategy, weakening immune suppression loops and restoring antitumor immunity.

Collectively, these combinatorial approaches underscore a central principle: targeting NAT10 does not simply inhibit a single oncogenic pathway but destabilizes an adaptive network that underlies multimodal therapy resistance. By pairing NAT10 inhibition with chemotherapy, anti-angiogenic therapy, or immunotherapy, it may be possible to dismantle the coordinated survival architecture that sustains advanced cancers.

### 7.3 Biomarker potential and clinical stratification

For NAT10-targeted strategies to achieve meaningful clinical impact, robust predictive biomarkers will be essential. Given NAT10’s role as an upstream integrator of metabolic, immune, and repair pathways, its expression and activity may reflect a broader adaptive tumor state rather than a single molecular alteration. Therefore, biomarker development should focus not only on NAT10 abundance but also on functional signatures that capture ac4C-driven network activation. Recent transcriptomic analyses have proposed the concept of an “ac4C modification score, ” derived from expression patterns of NAT10-regulated gene sets. Elevated ac4C-related signatures correlate with poor prognosis, enhanced glycolytic flux, and immunosuppressive phenotypes across multiple cancer types. Quantification of global ac4C levels or NAT10 expression may thus serve as a surrogate indicator of adaptive tumor programs characterized by metabolic plasticity and therapy resistance. Importantly, such signatures move beyond single-gene biomarkers and instead reflect epitranscriptomic network activity.

Because NAT10 intersects with multiple resistance pathways—including DNA damage repair, glycolysis, angiogenesis, and immune regulation—composite predictive models may provide greater clinical utility. Tumors exhibiting high NAT10 expression in conjunction with elevated DNA repair signatures may be predisposed to chemoresistance, whereas NAT10-high tumors enriched for glycolytic programs may exhibit immune exclusion phenotypes (128). Similarly, co-activation of NAT10 and VEGFA signaling may predict resistance to immune checkpoint blockade through vascular-mediated immune suppression. Integrating these axes into multidimensional predictive frameworks could enable rational patient stratification for combination therapies. Future clinical translation will require standardized and reproducible methods for measuring NAT10 activity and ac4C abundance. Prospective trials incorporating NAT10 expression, ac4C signatures, and multi-omics profiling—including transcriptomic, metabolomic, and immune landscape analyses—will be critical for validating its predictive value. Ultimately, incorporating NAT10 status into therapeutic decision-making may allow clinicians to identify patients most likely to benefit from epitranscriptomic intervention and rational combination strategies, advancing precision oncology beyond mutation-centric paradigms.

## 8 Integrated model: NAT10 as a central adaptive node

The accumulating evidence reviewed above converges on a unifying conceptual framework: NAT10 operates as a central adaptive node that integrates environmental stress signals with epitranscriptomic remodeling to sustain malignant progression. Rather than functioning as a linear effector downstream of oncogenic signaling, NAT10 sits at the intersection of transcriptional activation, RNA modification, metabolic control, and microenvironmental regulation. At the upstream level, diverse stress inputs—including hypoxia, mechanical signaling (YAP1 activation), inflammatory stimuli, metabolic imbalance, and proteostatic alterations—converge to enhance NAT10 expression, stability, or spatial organization. These inputs do not merely increase NAT10 abundance; they recalibrate its catalytic output and substrate engagement, positioning NAT10 as a stress-responsive epitranscriptomic amplifier.

Upon activation, NAT10 reshapes the transcriptome through ac4C deposition on selected mRNAs and tRNAs. This remodeling does not globally alter RNA metabolism; instead, it preferentially stabilizes transcripts encoding metabolic enzymes, DNA repair factors, adhesion molecules, immune modulators, and signaling intermediates. In parallel, NAT10-mediated tRNA acetylation enhances translational capacity, while chromatin-associated RNA interactions couple ac4C activity to transcriptional reinforcement. The result is a coordinated increase in protein output across interconnected oncogenic pathways. These molecular changes propagate into functional adaptation (18, 129). Simultaneously, vascular remodeling, immune suppression loops, and macrophage polarization reshape the tumor microenvironment. Through these mechanisms, NAT10 bridges intrinsic tumor cell programs with extrinsic ecosystem remodeling.

**Figure 6:**
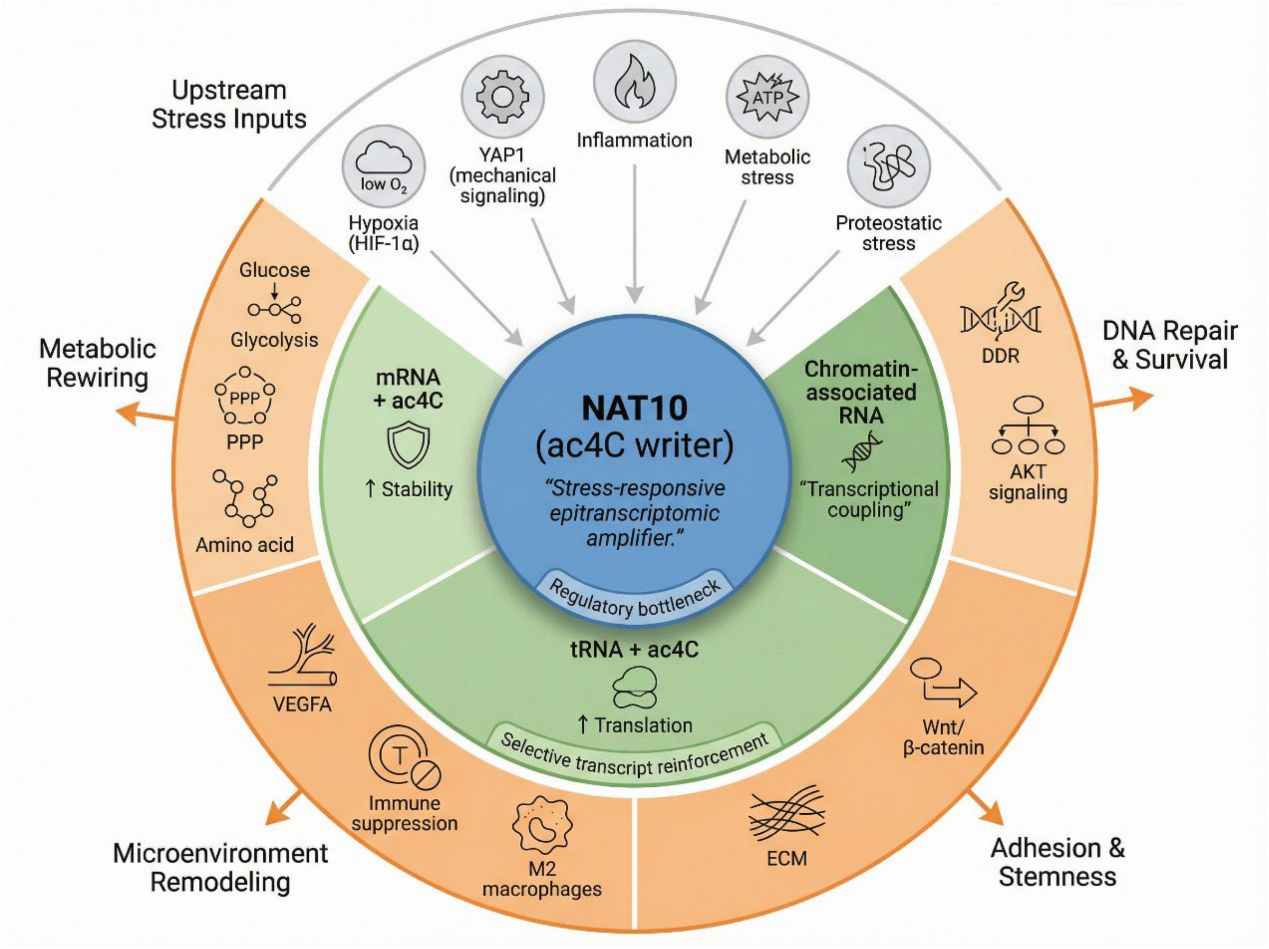
Integrated model of NAT10 as a central adaptive node. Diverse stress inputs—including hypoxia, YAP1 activation, inflammation, and metabolic imbalance—converge to activate NAT10. Through ac4C-mediated remodeling of selected mRNAs and tRNAs, NAT10 enhances transcript stability, translation, and chromatin-associated transcriptional reinforcement. These coordinated effects drive metabolic rewiring, DNA repair, immune suppression, and angiogenic signaling, forming self-reinforcing feedback circuits that sustain tumor plasticity and therapy resistance.

Importantly, these layers form self-reinforcing feedback circuits. Hypoxia enhances NAT10 activity, NAT10 stabilizes HIF-1α–dependent transcripts, and glycolytic flux sustains hypoxic signaling. Wnt/β-catenin signaling promotes NAT10 expression, while NAT10 stabilizes Wnt pathway components. Angiogenic signaling amplifies hypoxia, which further stimulates NAT10. These feedback loops transform NAT10 from a downstream modifier into a network amplifier capable of sustaining oncogenic states even in the presence of therapeutic intervention. Within this integrated model, tumor plasticity emerges as a product of epitranscriptomic reinforcement. By stabilizing adaptive transcripts and amplifying metabolic and immune escape programs, NAT10 enables cancer cells to transition between proliferative, invasive, and therapy-resistant states without requiring new genetic alterations. In this sense, NAT10 functions as a regulatory rheostat that tunes the amplitude and persistence of oncogenic signaling. As illustrated in **Figure 6**, NAT10 integrates environmental stress signals with ac4C-dependent transcript reinforcement to drive coordinated metabolic, immune, and survival adaptation.

## 9 Critical view and future perspectives

Despite rapid progress, the NAT10–ac4C field remains in an early, conceptually fluid stage. Current studies collectively support NAT10 as a central adaptive node, yet several key uncertainties limit mechanistic generalization and clinical translation. Below we highlight critical gaps, potential biases, and future directions that—if addressed—could transform NAT10 biology from an emerging framework into a mature therapeutic paradigm.

### 9.1 Evidence landscape: strengths, biases, and generalizability

A notable limitation of the current literature is cancer-type and pathway bias. Most mechanistic evidence is concentrated in a subset of tumor types (e.g., gastric, bladder, prostate, breast, pancreatic cancers and AML), often with an emphasis on metabolic rewiring, hypoxia adaptation, and immune suppression (130, 131). Whether NAT10 plays comparable roles in other malignancies (e.g., brain tumors, melanoma, ovarian cancer) remains underexplored. Moreover, many studies focus on NAT10 overexpression or knockdown phenotypes, which may overestimate linear causality. A key unanswered question is whether NAT10 is universally oncogenic or context-dependent—potentially acting as a vulnerability in certain molecular subtypes but dispensable in others. A second concern is model-system dependence. Many conclusions rely on established cell lines, xenografts, or single-model readouts. These systems may not faithfully capture human tumor heterogeneity, immune complexity, or therapeutic selection pressures. In particular, claims about immune modulation often require robust validation in immunocompetent or humanized models, alongside spatial and single-cell profiling of immune states. Key unresolved questions and future research priorities in NAT10 biology are outlined in **Table 3**.

**Table 3:** Outstanding questions and future directions in NAT10 biology.

Key question	Current limitation	Proposed solution
Cancer-type bias?	Limited tumor spectrum studied	Pan-cancer multi-omics analysis
ac4C detection reliability?	Antibody and mapping variability	Quantitative, stoichiometric ac4C sequencing
Are there ac4C readers?	No definitive reader identified	RNA pulldown + proteomics screens
mRNA vs tRNA contribution?	Relative impact unclear	Substrate-specific genetic models
Tissue specificity?	Context dependence not defined	Single-cell spatial profiling
Non-enzymatic roles?	Catalytic vs scaffolding unclear	Catalytic-dead vs degrader studies
Therapeutic targeting strategy?	Remodelin limitations	PROTACs, allosteric inhibitors
Clinical stratification?	No standardized ac4C scoring	Prospective biomarker-driven trials

### 9.2 Physiological functions of NAT10 and therapeutic safety considerations

Beyond its oncogenic functions, NAT10 has essential roles under physiological conditions. NAT10 was initially characterized as an ATP-dependent RNA acetyltransferase required for ac4C formation in 18S rRNA, pre-18S rRNA processing, and 40S ribosomal subunit biogenesis (45). NAT10-related ac4C-modifying machinery also contributes to tRNA acetylation, translational fidelity, and stress-responsive translational control, indicating that NAT10 supports basal RNA metabolism and proteome maintenance rather than functioning exclusively in malignant cells [PMID: 40846426]. In addition, NAT10 has been implicated in DNA damage responses and nuclear architecture, as DNA damage can induce NAT10 expression and pharmacological NAT10 inhibition by Remodelin can correct laminopathic nuclear abnormalities (78). More recently, NAT10-mediated mRNA ac4C has been linked to physiological developmental processes, including translational regulation during mouse oocyte meiotic maturation (132). These observations indicate that NAT10 is not a tumor-specific enzyme but a broadly acting regulator of RNA metabolism, translation, genome stress responses, and cellular architecture under non-malignant conditions. Therefore, systemic NAT10 inhibition may affect normal proliferative or stress-responsive tissues, including hematopoietic cells, intestinal epithelium, germ cells, and immune compartments. Future studies should define tumor-specific NAT10 dependency relative to normal tissues, clarify the therapeutic window, and evaluate whether selective targeting can be achieved through biomarker-defined tumors, optimized dosing schedules, or combination strategies that reduce the required degree of NAT10 inhibition.

### 9.3 Technical caveats: measuring ac4C in the transcriptome

The field is also constrained by methodological limitations in ac4C detection and quantification. Compared with m6A, ac4C mapping technologies are less standardized, and different platforms can produce variable site calls due to antibody specificity, chemical derivatization biases, and dependence on RNA structure. Many datasets provide relative enrichment rather than absolute stoichiometry, making it difficult to distinguish biologically meaningful ac4C from low-level background modification. Additionally, ac4C sites can be sensitive to RNA folding and sequence context, raising concerns about false positives/negatives across experimental conditions. A critical future requirement is the development of quantitative, reproducible, and cross-platform validated ac4C mapping, ideally with internal standards, stoichiometry estimation, and orthogonal validation (e.g., site-specific mass spectrometry). Without this, translational biomarker efforts—such as “ac4C scores”—risk being correlative rather than mechanistically anchored.

### 9.4 The missing layer: readers, effectors, and mechanistic specificity

A major conceptual gap is whether ac4C has dedicated reader proteins analogous to m6A readers (YTH family). Many current models implicitly assume that ac4C exerts direct physicochemical effects on RNA stability and translation, which is plausible, but does not explain target selectivity, context dependence, and pathway-level coordination. It remains unclear whether ac4C recruits specific RNA-binding proteins, alters RNP assembly, or changes ribosome dynamics in a transcript-specific manner. In parallel, the relative contribution of tRNA versus mRNA acetylation remains unresolved. Several studies highlight tRNA-mediated translational effects, while others focus on mRNA stability and translation. Determining which RNA substrate class is dominant—and under what conditions—will be essential for therapeutic targeting. It is possible that mRNA ac4C drives transcript-specific oncogenic programs, whereas tRNA ac4C supports global translational resilience, with both contributing differently across tumor contexts and stress states.

### 9.5 Context dependence: tissue specificity and non-enzymatic functions

Another underappreciated challenge is tissue specificity. NAT10 operates within diverse cellular environments with different transcriptional programs, metabolic baselines, and immune landscapes. Thus, the same NAT10-driven ac4C event could produce distinct biological outputs depending on tissue type, differentiation state, or microenvironmental stress. Future studies must distinguish universal NAT10 programs from context-specific modules, ideally through comparative multi-tumor atlases. Finally, it remains unclear to what extent NAT10’s oncogenic activity depends strictly on catalytic ac4C deposition versus non-enzymatic scaffolding functions. Evidence of NAT10 involvement in phase-separated condensates, chromatin-associated RNA regulation, and protein–protein interactions suggests that NAT10 may function as a structural organizer of RNA-processing hubs (133, 134). If non-enzymatic roles are substantial, purely catalytic inhibitors may be insufficient—supporting the need for degraders or strategies that disrupt NAT10 localization and condensate dynamics.

Tumor-specific roles of NAT10 should be interpreted within a context-dependent framework. Although NAT10 overexpression is frequently associated with malignant progression, metabolic adaptation, and therapy resistance, its downstream outputs may vary substantially across cancer types. In glycolysis-dependent tumors such as gastric cancer, non-small cell lung cancer, ovarian cancer, and retinoblastoma, NAT10 appears to reinforce metabolic enzymes and glycolytic flux, thereby supporting proliferation and stress adaptation (14, 15, 18). In colorectal and prostate cancer, NAT10 has been linked more prominently to Wnt/β-catenin signaling, cytoskeletal remodeling, epithelial–mesenchymal transition, and metastatic dissemination (30, 38). In pancreatic cancer, NAT10-related programs have been associated with AXL- or LAMB3-mediated tumor progression, stromal interaction, and immune microenvironmental remodeling, whereas in clear-cell renal cell carcinoma, NAT10-mediated ac4C modification of ANKZF1 promotes YAP1-associated tumor progression and lymphangiogenesis (13, 32, 97). In hematologic malignancies such as acute myeloid leukemia, NAT10 may preferentially regulate amino acid metabolism, stemness-associated programs, and leukemic cell survival (67, 130). These observations suggest that NAT10 does not drive a single universal oncogenic pathway; instead, it may amplify the dominant adaptive program required by a given tumor lineage or microenvironmental state. Therefore, future studies should define tumor-type-specific NAT10 dependencies, distinguish shared ac4C-regulated modules from lineage-restricted targets, and determine whether NAT10 inhibition produces differential therapeutic windows across cancer types.

### 9.6 Future directions: toward a mature translational framework

#### 9.6.1 Single-cell and spatial ac4C mapping

To resolve heterogeneity and tissue specificity, the next frontier will be single-cell and spatially resolved ac4C profiling, integrated with transcriptomics, metabolomics, and immune phenotyping. This will clarify which cell populations (tumor cells vs stromal vs immune) exhibit NAT10-driven ac4C programs and how these evolve during treatment.

#### 9.6.2 Identification of ac4C readers and effectors

Systematic discovery platforms—RNA pulldown proteomics, CRISPR screens for ac4C-dependent phenotypes, and structural studies—should be prioritized to identify ac4C readers or effector complexes. Defining the reader layer would greatly improve mechanistic specificity and enable drug discovery beyond NAT10 itself (135).

#### 9.6.3 Development of NAT10 degraders and next-generation inhibitors

Given potential non-enzymatic functions and proteostasis-dependent regulation, NAT10 degraders (e.g., PROTACs or molecular glues) may provide broader suppression than catalytic inhibition alone. Parallel development of selective, drug-like NAT10 inhibitors remains critical, including allosteric modulators and RNA-binding interface disruptors.

#### 9.6.4 Targeting NAT10 condensates and spatial regulation

If phase separation proves essential for NAT10 function, targeting condensate formation or stability could represent a novel therapeutic strategy. This will require mechanistic dissection of condensate composition, biophysical determinants, and stress-dependence, alongside pharmacologic tools to perturb LLPS.

#### 9.6.5 Metabolism–immune combinatorial therapy

Because NAT10 simultaneously controls metabolic flux and immune suppression loops, rational combinations should focus on metabolism–immunity coupling. Future trials could explore NAT10 inhibition together with immune checkpoint blockade, anti-angiogenic agents, or metabolic inhibitors (e.g., glycolysis/PPP axis blockers), guided by biomarker-defined patient stratification.

## 10 Conclusion

The past few years have witnessed a rapid expansion in our understanding of NAT10 and its catalytic product, N4-acetylcytidine (ac4C), revealing a regulatory axis that extends far beyond conventional RNA modification. What began as the characterization of an RNA acetyltransferase involved in ribosomal and tRNA processing has evolved into the recognition of NAT10 as a central coordinator of tumor adaptation. Through selective ac4C deposition on mRNA and tRNA, NAT10 amplifies oncogenic transcript stability, enhances translational efficiency, and reinforces metabolic and immune programs that sustain malignant progression. Across diverse cancer types, NAT10-mediated epitranscriptomic remodeling converges on several core adaptive modules: metabolic reprogramming, DNA damage repair, drug efflux, vascular remodeling, immune suppression, and metastatic niche formation. Importantly, these outputs are not independent; they form interconnected feedback circuits that stabilize tumor plasticity under stress conditions such as hypoxia, nutrient limitation, and therapeutic pressure. In this context, NAT10 functions not merely as a downstream effector but as a regulatory hub that integrates environmental signals with transcriptomic execution, enabling tumors to dynamically shift between proliferative, invasive, and therapy-resistant states without requiring new genetic alterations. From a translational perspective, targeting NAT10 offers an opportunity to disrupt adaptive resilience at its regulatory root. By attenuating ac4C-dependent stabilization of key transcripts, NAT10 inhibition has the potential to sensitize tumors to chemotherapy, overcome targeted therapy resistance, normalize aberrant vasculature, and enhance immune checkpoint responsiveness. At the same time, the development of selective inhibitors, degraders, and biomarker-guided strategies will be essential to harness this potential safely and effectively.

Despite these advances, the field remains in a formative stage. Key questions—such as the identification of ac4C readers, the relative contributions of mRNA versus tRNA acetylation, the role of phase separation, and the context specificity of NAT10 programs—must be resolved to fully define its biological scope. Future integration of single-cell epitranscriptomics, spatial tumor ecology, and multi-omics profiling will be critical to refine mechanistic understanding and clinical stratification. In summary, NAT10 represents a paradigm shift in cancer biology: an epitranscriptomic integrator that links RNA regulation, metabolic plasticity, and microenvironmental remodeling into a coherent adaptive network. Continued investigation into this central node may not only deepen our understanding of tumor adaptability but also open new avenues for overcoming therapeutic resistance in precision oncology.
